# The effects of HAP and macrophage cells to the expression of inflammatory factors and apoptosis in HK-2 cells of vitro co-cultured system

**DOI:** 10.1007/s00240-017-1032-8

**Published:** 2017-12-13

**Authors:** Junchuan Yu, Yaoliang Deng, Zhiwei Tao, Weixia Liang, Xiaofeng Guan, Jihua Wu, Xin Ning, Yunlong Liu, Quan Liu, Ziqi He

**Affiliations:** Department of Urology, The First Affiliated Hospital of Guangxi Medical University, Nanning, 530021 China

**Keywords:** Macrophage cells, Hydroxyapatite, Renal tubular epithelial cells, Co-culture, Fetuin-A

## Abstract

This study developed an in vitro system by co-culturing HK-2 cells with different concentration of hydroxyapatite (HAP) and/or macrophage cells to simulate the internal environment of urolithiasis as far as possible, investigating the regulatory effects of macrophage cells on HAP-induced expression of relative inflammatory factors of HK-2 cells. The control group (H group) was only comprised of HK-2 cells. Experimental groups included co-culturing HK-2 cells and macrophage cells (H + M group), co-culturing HK-2 cells and HAP (H + A group), co-culturing macrophage cells and HAP (M + A group), and co-culturing HK-2 cells and macrophage cells with HAP (H + M + A group). In the H + A, M + A, and H + M + A group, we set the concentration of HAP as 5 μg/cm^2^ (A1) and 10 μg/cm^2^ (A2). After co-culturing for 2, 4, and 6 h, we detected the expression of CCL-2 in the liquid by ELISA. We tested the expression of LDH and ROS to evaluate the damage of HK-2 cells. We assessed the apoptosis of HK-2 cells using DAPI staining assay, flow cytometry, and the rate of BAX/BCL-2. Western Blotting detected OPN, Fetuin-A, BAX, and BCL-2 of HK-2 cells. The expression of CCL-2 in the medium of H + A1 and H + A2 group increased significantly compared with the control (*P* < 0.05); CCL-2 of M + A1 and M + A2 group was higher than the H + A1 and H + A2 group (*P* < 0.05). The expression of CCL-2 in H + M + A1 and H + M + A2 group was also higher than M + A1 and M + A2 group (*P* < 0.05). Compared with control, the expression of OPN, LDH release, the ratio of BAX/BCL-2, and the generation of ROS in HK-2 cells increased in a dose- and time-dependent manner. Compared with the control, the expression of Fetuin-A decreased in various degrees at different incubation periods. Especially when co-culturing for 6 h, Fetuin-A decreased most seriously in the H + M + A1 group. (1) The HAP can induce the HK-2 cells oxidative stress and inflammatory damage and apoptosis, when adding the macrophages to co-culture, macrophage cells can aggravate the damage and apoptosis of the HK-2 cells. (2) After the stimulation of HAP, the expression of OPN in HK-2 cells increased in a time- and dose-dependent manner; macrophage cells can aggravate the increase of OPN in HK-2 cells. (3) In the HAP and HK-2 cells co-cultured system, the low-level Fetuin-A of HK-2 cells may be related to the excessive consumption of Fetuin-A in the process of HAP-induced renal tubular epithelial cell excessive oxidative stress, inflammatory injury, and cell apoptosis. When adding macrophage cells to co-culture, Fetuin-A decreased even more seriously, it reminds us that macrophage cells can slightly regulate the expression of Fetuin-A in the HK-2 cells.

**Electronic supplementary material:**

The online version of this article (10.1007/s00240-017-1032-8) contains supplementary material, which is available to authorized users.

## Introduction

Urolithiasis is a common disease in urology; about 5–15% of people worldwide suffer from urolithiasis [1]. The recurrent urolithiasis can cause significant economic and medical implications [2], it also leads to a series of complications, such as metabolic syndrome, and chronic and terminal kidney diseases [3, 4]. To explore the etiology and pathogenesis of urinary calculi has always been the hot spot of the urology research, at present, we generally believed that the interaction of genetic, diet, environment, and calcium metabolism abnormality participated in the formation and development of urolithiasis, the abnormal calcium metabolism includes the mineral heterogeneity nucleation, crystal growth aggregation, and the adhesion of the renal epithelial cells [5, 6]. The most common stone in urolithiasis is calcium oxalate [7]. However, the formation mechanism of calcium oxalate calculi is still unknown.

Alexander Randall regraded calcium phosphate plaques in renal papillae as the origin of kidney stones [8]. The plaques which we called Randall’s plaques (RP) originate deep inside the renal interstitium associated with the basement membranes of loops of Henle, and can promote the nucleation, growth, and aggregation of CaO_*x*_ crystals in renal epithelial cells [8]. Recent studies evidence that the number of calcium oxalate stone is proportional to the covered area of RP [9], but the main mineral phase of RP is seems to be irrelevant to calcium oxalate stones in the form of hydroxyapatite [10], so there is an unavoidable connection between hydroxyapatite and calcium oxalate stone. The release of inflammatory reaction caused by the infiltration of macrophages in the intercellular space of the crystals and the release of chemokine CCL-2 was also found to promote the formation of stones [11–14]. Macrophages can alter their function based on the activation program utilized—either M1 or M2 patterns [15]. M1 macrophages are thought to be antitumorigenic as well as be pro-inflammatory and antimicrobial, although this remains the subject of debate [15]. M2 macrophages are associated with wound healing and have pro-tumorigenic properties [15, 16]. The inflammation which triggered by macrophage cells infiltration of the intercellular space around the crystals is also found to be the important process of stone formation [11]. C-C motif chemokine ligand 2 (CCL-2) is a chemokine and osteopontin (OPN) are also beneficial to urolithiasis [12–14]. Adhesion effect of OPN may inspire the process of stone crystal heterogeneous nucleation, and promote the formation of stones. Renal epithelial cell injury and apoptosis facilitates crystal adhesion to cell surface, largely, which is a key step in urolithiasis [17, 18]. When the cells are damaged, they release a large amount of reactive oxygen species (ROS), which is some intermediate metabolite of oxygen or the derivative of oxygen; ROS has more oxidative capacity than oxygen. Under normal conditions, the ROS level is very low and does not cause harm. The generation and removal of reactive oxygen in cells is in a dynamic equilibrium state. Once this balance is broken, the damage can be done, causing oxidative stress damage to the cells and death of the cells in severe cases [19], the basal membrane of the damaged renal tubular epithelial cells was exposed, making it easier for more crystals to adhere to the renal tubular epithelial cells, resulting in calcium deposition. Fetuin-A, also known as a2-Heremans–Schmid glycoprotein (AHSG), possesses potent calcification-inhibitory activity [20]. It had been proved that patients with urolithiasis had lower urine Fetuin-A levels compared with the control [21].

To sum up, since the Fetuin-A, hydroxyapatite, macrophages, and cell apoptosis are associated with the formation of urinary stones, so we venture to guess that whether there is some kind of connection among them. To test our hypothesis, we developed an in vitro system by co-culturing HK-2 cells with different concentration of HAP and/or macrophage cells to close to the internal environment of urolithiasis as far as possible, this co-culture can make up for the information defect caused by the previous single-cell plant research, so as to make the body match the environment in vitro as far as possible. Therefore, we can better explore the role of macrophages–renal tubular epithelial cell–hydroxyapatite in RP formation, clarify the relationship between the various inflammatory factors related, and may provide a new idea for the mechanism of RP formation.

## Materials and methods

### Cell culture

HK-2 cells were purchased from China Center for Type Culture Collection and cultured in DMEM/F12 (Life Technologies^™^) supplemented with 10%FBS (Gibcol, from Life Technologies^TM^),100 U/ml penicillin and 100 mg/ml streptomycin in a humidified 5% CO_2_ incubator at 37 °C. We routinely seeded cells at a density of 5 × 10^4^/cm^2^ in culture vessel (Corning, Life Technologies^™^). We changed medium every day and subcultured cells before forming confluent monolayers.

The human monocytic cell line U937 were obtained from China Center for Type Culture Collection and grown in RPMI 1640 BASIC (Life Technologies^™^) supplemented with 10% FBS. We transferred the cell suspension into a tube and centrifuged cells at 1000 r.p.m. × 5 min. Thus, the cells were washed and suspended in RPMI1640 BASIC (Life Technologies^™^) with 10% FBS for use. Then, cell density was adjusted to 1 × 10^6^ cells/ml in RPMI1640 BASIC containing 10% FBS, and the cells were distributed in the isolated upper compartments of a transwell system and incubated for 1 day in a humidified 5% CO_2_ incubator at 37 °C. We treated the cells with 80 ng/ml phorbol 12-myristate 13-acetate (PMA, from Sigma-Aldrich) for 48 h at 37 °C and named as Mφ macrophage cells. Then, for M1 macrophage differentiation, the Mφ macrophage cells were stimulated by RPMI1640 BASIC with 500 ng/ml LPS (O55:B5, Sigma-Aldrich), and for M2 macrophage differentiation, the Mφ macrophage cells were stimulated by RPMI1640 BASIC with 20 ng/ml IL-4 (SRP3093, Sigma-Aldrich).

### Groups and co-culturing

**Fig. 1:**
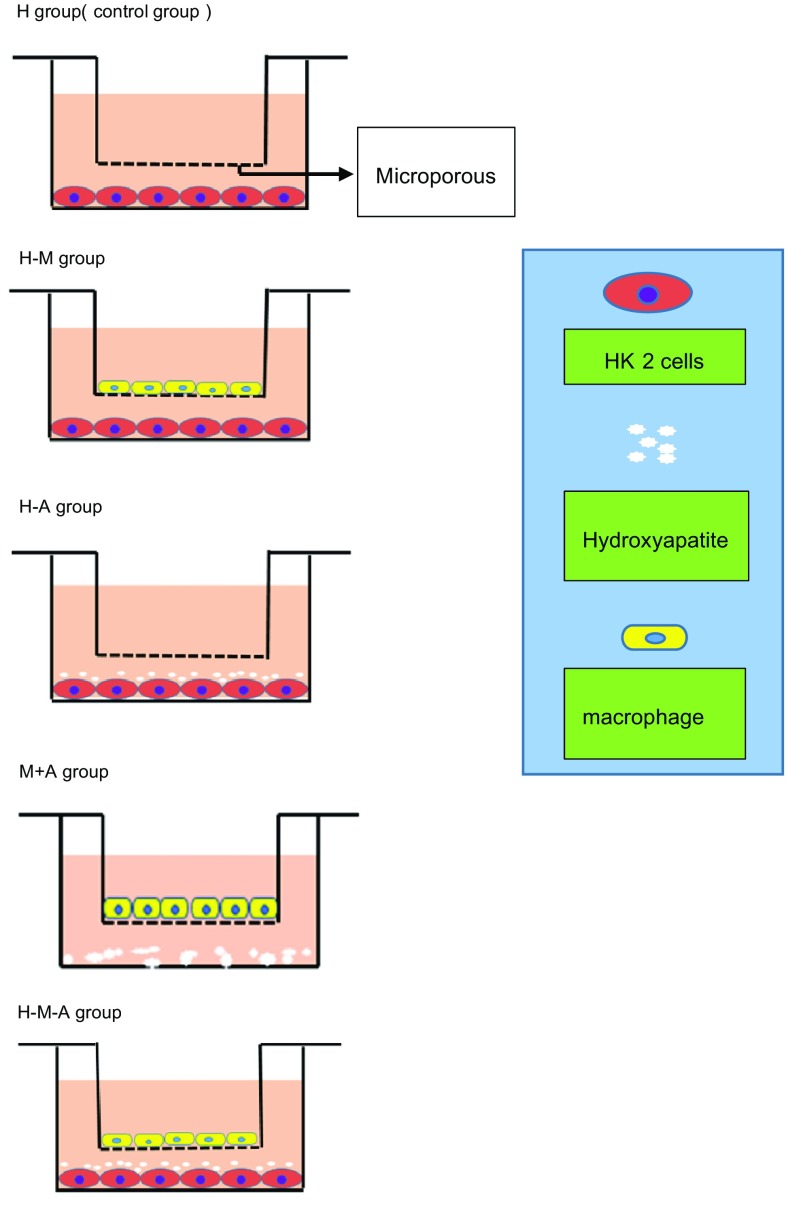
In co-cultured system, three groups were established, including H (control)—HK-2 cells cultivated independently of other types, H + M—co-cultured HK-2 cells and M1 macrophage cells, H + A—co-cultured HK-2 cells and HAP, M + A—co-cultured M1 macrophage cells and HAP, H + M + A—co-cultured HK-2 cells, HAP and M1 macrophage cells. Microporous membranes separated upper and lower compartments

Six groups were established. The H group (controls) was only comprised of HK-2 cells. Co-culture groups included H + M group (HK-2 cells co-cultured with macrophage cells), H + A group [HK-2 cells co-cultured with HAP (677418, from Sigma-Aldrich)], and H + M + A group (HK-2 cells co-cultured with HAP and macrophage cells). We carried out co-culture in a new transwell system. First, we differentiated macrophage cells in the isolated upper compartments. We changed medium into DMEM/F12 without FBS before co-culturing. We seeded HK-2 cells in the lower compartment, and replaced medium with FBS-free medium before co-culturing. Based on relative study [22], in the H + A group and the H + M + A group, we, respectively, transferred HAP into the lower compartment with the concentration of 5 and 10 μg/cm^2^ (Fig. 1). After co-culturing for 2, 4 and 6 h, we, respectively, investigate lactate dehydrogenase (LDH) level, reactive oxygen species (ROS) level, 4′-6-diamidino-2-phenylindole (DAPI) staining assay kit, Enzyme-linked immunosorbent assays (ELISA), Flow Cytometry, and Western Blotting. In addition, when testing the expression of CCL-2 in the medium, we added the M + A group (macrophage cells co-cultured with HAP), we, respectively, transferred HAP into the lower compartment with the concentration of 5 and 10 μg/cm^2^ (Fig. 1).

### Lactate dehydrogenase (LDH)

As an indication of cell injury, we measured LDH release from the HK-2 cells of all groups in the media. Media of all groups were aliquoted to 96-well plates; then, LDH activity was assayed by absorbance change at a wavelength of 450 nm on a Multiskan^™^ GO microplate reader (Thermo Scientific^™^) with assay kit (Nanjing Jiancheng Bioengineering Institute, Nanjing, China).

### Flow cytometry

To determine whether the reduced cell viability is related to apoptosis, flow cytometry analysis was further performed using an Annexin V-Fluorescein Isothiocyanate (FITC) Apoptosis Detection kit (BD Pharmingen, San Diego, CA, USA), according to the manufacturers protocol.

After being co-cultured for 2, 4, and 6 h, the cells were then washed twice with PBS, centrifuged at 375×*g* for 5 min, and resuspended in 500 µl binding buffer. We added 5 µl Annexin V-FITC and mixed into the cell suspension, followed by the addition of 5 µl propidium iodide and subsequent mixing, and then incubated the reaction for 5–15 min in a dark room. Finally, we detected the early apoptotic cells by flow cytometry within 1 h.

### Western blotting

After co-culturing for 2, 4, and 6 h, total protein was, respectively, extracted from cultured HK-2 cells only in H, H + A and H + M + A group using the RIPA Lysis Buffer (Beyotime) containing 2% PMSF (Beyotime) according to the manufacturer’s instructions, and then collected protein samples and subjected to western blotting performed essentially according to an established procedure [23]. The primary antibodies used were as follows: mouse anti-human GAPDH (1:10,000, ProteinTech, Chicago, IL, USA), mouse anti-human Fetuin-A, and Anti-Osteopontin (1:2000, Abcam, Cambridge, MA, USA), and BCL-2 (1:2000, D55G8) Rabbit mAb, BAX Antibody (1:2000). We performed quantifications by measuring band intensities using AlphaView and Image lab analysis software.

### 4′-6-diamidino-2-phenylindole staining assay

The apoptotic nuclei were detected using 4′-6-diamidino-2-phenylindole (DAPI) assay kit (Solarbio Corporation, Beijing, China), which is a deoxyribonucleic acid-specific fluorescent dye, as previously described [24].

### Determination of reactive oxygen species (ROS) generation

We measured ROS generation with the fluoroprobe, 2′, 7′-dichlorodihydrofluorescein diacetate (DCF-DA) (Solarbio Corporation, Beijing, China). After the cells were co-cultured for 2, 4, and 6 h, the cells were incubated with 5 μM DCF-DA for 30 min at 37 °C, as previously described [25]. We obtained the images using a fluorescence microscope (Olympus Corporation, Tokyo, Japan).

### Cytokine measurement in culture medium

We performed enzyme-linked immunosorbent assays to measure levels of CCL-2, TNF-α, and TNF-β (R&D Systems; Minneapolis, MN, USA) produced in the supernatant of each culture dish.

### Statistical analysis

One-way analysis of variance (ANOVA). We performed the statistical analyses using statistical software (GraphPad Software Inc., San Diego, CA, USA). We considered a *P* < 0.05 as statistically significant.

## Results

### The induction and qualification of M1 macrophage cells

**Fig. 2:**
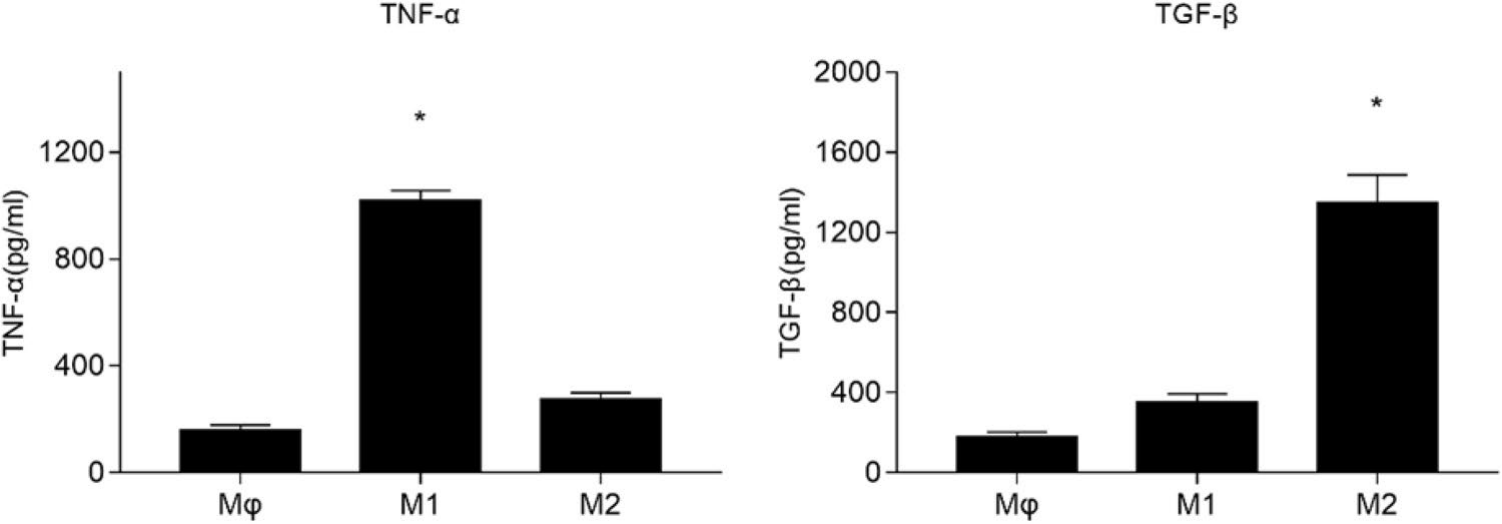
Expression of TNF-α and TGF-β in the media. Each column represented the mean ± the standard error of the mean. *P < 0.05 vs. Mφ. ^#^ *P* < 0.05 vs. Mφ. The data were representative of at least three independent experiments

Based on the mentioned methods, we induced the U937 cells to M1 and M2 macrophage cells. As TNF-α is a typical M1-marker and TGF-β is a typical M2-marker [26, 27], we detected, respectively, the expression of TNF-α and TGF-β in the medium by ELISA to evaluate whether we successfully induced the M1 macrophage cells or not. Like Fig. 2 showed, TNF-α in the medium of M1 macrophage cells was higher than Mφ and M2 (*P < 0.05), TGF-β in the medium of M2 macrophage cells was higher than Mφ and M1 (^#^ *P* < 0.05). These results demonstrated that we successfully induced the M1 macrophage cells (Fig. 2).

### The expression of CCL-2 in the medium

At different co-culture time, CCL-2 in the medium was no statistically different between the H + M group and the H group (*P* > 0.05). It demonstrated that the HK-2 cells could not activate macrophage cells, although the HK-2 and U937 cells originated from different donors. In other words, due to the HK-2 cells and U937 cells came from different donors and HK-2 cells may have certain immunogenicity of macrophage cells, but this kind of immunogenicity was not enough to activate macrophages to come up with severe inflammation reaction, and the interference to our experimental results can be neglected.

**Fig. 3:**
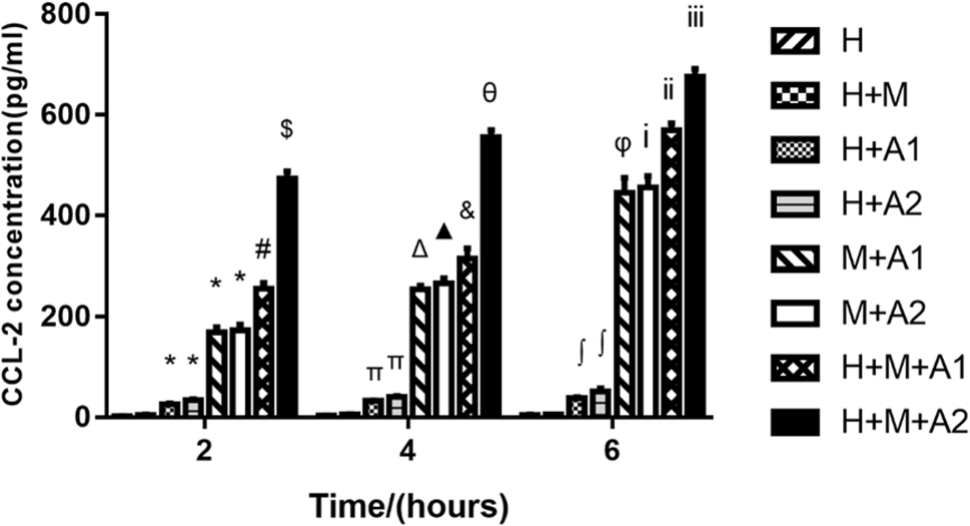
Expression of CCL-2 in the medium of the H, H + M, H + A1, H + A2, M + A1, M + A2, H + M + A1, and H + M + A2 groups at 2, 4, and 6 h, respectively. Each column represented the mean ± the standard error of the mean. *P < 0.05 vs. H group (2 h). ^#^ *P* < 0.05 vs. H group (2 h), H + A1 group (2 h) and M + A1 group (2 h). ^$^ *P* < 0.05 vs. H group (2 h), H + A2 group (2 h), M + A2 group (2 h), H + M + A1 group (2 h). Δ*P* < 0.05 vs. H group (4 h), M + A1 group (2 h). ^▲^ *P* < 0.05 vs. H group (4 h), M + A2 group (2 h). ^&^ *P* < 0.05 vs. H group (4 h), H + A1 group (4 h), M + A1 group (4 h) and H + M + A1 group (2 h). θ*P* < 0.05 vs. H group (4 h), H + A2 group (4 h), M + A2 group (4 h), H + M + A2 group (2 h) and H + M + A1 group (4 h). ^φ^ *P* < 0.05 vs. H group (6 h), M + A1 group (2 h), M + A1 group (4 h). ^i^P < 0.05 vs. H group (6 h), M + A2 group (2 h) and M + A2 group (4 h). ^ii^P < 0.05 vs. H group (6 h), H + A1 group (6 h), M + A1 group (6 h), H + M + A1 group (2 h), and H + M + A1 group (4 h). ^iii^P < 0.05 vs. H group (6 h), H + A2 group (6 h), M + A2 group (6 h), H + M + A1 group (6 h), H + M + A2 group (2 h), and H + M + A2 group (4 h). The data were representative of at least three independent experiments

The CCL-2 in the medium of H + A and H + A2 increased statistically compared with the control (*P* < 0.05). We can infer that HAP can activate the HK-2 cells to secrete a small amount of CCL-2. However, compared with M + A1 and H + A1 group in our observation window, we found that the CCL-2 was mainly from the macrophage cells. In addition, the concentration of HAP was higher and the stimulation time longer, the expression of CCL-2 increased, and the inflammation aggravated. If we co-cultured HK-2 cells with HAP and macrophage cells, the expression of CCL-2 can be even higher. For example, compared with H, H + A1 and M + A1 group, the CCL-2 of H + M + A1 group increased statistically (*P* < 0.05). Moreover, the CCL-2 of H + M + A2 group went up statistically in a time-dependent manner compared with the H + M + A1 group (*P* < 0.05) (Fig. 3).

The above results show that the CCL-2 are mainly from the M1 macrophage cells, although HAP also can stimulate the HK-2 cells to produce a certain amount of CCL-2. HAP could induce HK-2 cells to inflammation in a time-dependent manner. In addition, the M1 macrophage cells can aggravate this inflammation.

### The expression of OPN in HK-2 cells

**Fig. 4:**
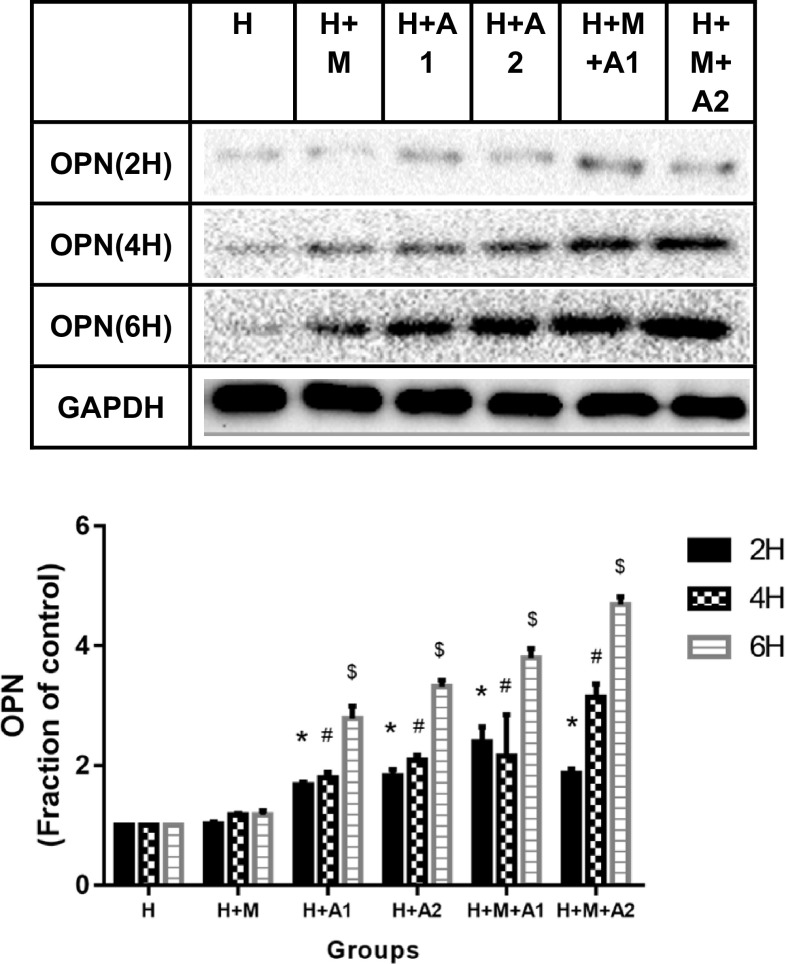
Expression of OPN in HK-2 cells of all groups at 2, 4, and 6 h, respectively. Each column represented the mean ± the standard error of the mean. *P < 0.05 vs. H group, 2 h. ^#^ *P* < 0.05 vs. H group, 4 h. ^$^ *P* < 0.05 vs. H group, 6 h. The data were representative of at least three independent experiments

At 2 h, the osteopontin (OPN) protein in HK-2 cells increased significantly in the H + A1, H + A2, H + M + A1, and H + M + A2 group (*P* < 0.05 vs. H group, 2 h). At 4 and 6 h, there were significantly increase from the H + A1 group to the H + M + A2 group (*P* < 0.05). These results can fully prove that the expression of OPN in HK-2 cells increased in a dose-and time-dependent manner after the stimulation of HAP; meanwhile, the M1 macrophage cells can lead the OPN in HK-2 cells to increase much more. In our observation window, the expression of OPN in H + M + A2 group was the highest (Fig. 4).

### Reactive oxygen species generation (ROS)

**Fig. 5:**
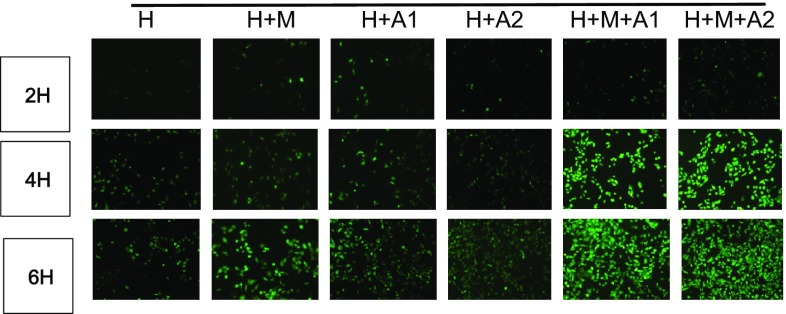
Original magnification, ×10. Scale bar 50 μm

We detected the generation of ROS using the ROS-sensitive fluorescent dye, DCF-DA. The intensity of DCF-DA fluorescence increased in a dose-and time-dependent manner (*P* < 0.05). In our observation window, the intensity of DCF-DA in H + M + A2 group at 6 h incubation time achieved the highest (Fig. 5).

### Lactate dehydrogenase (LDH) release

**Fig. 6:**
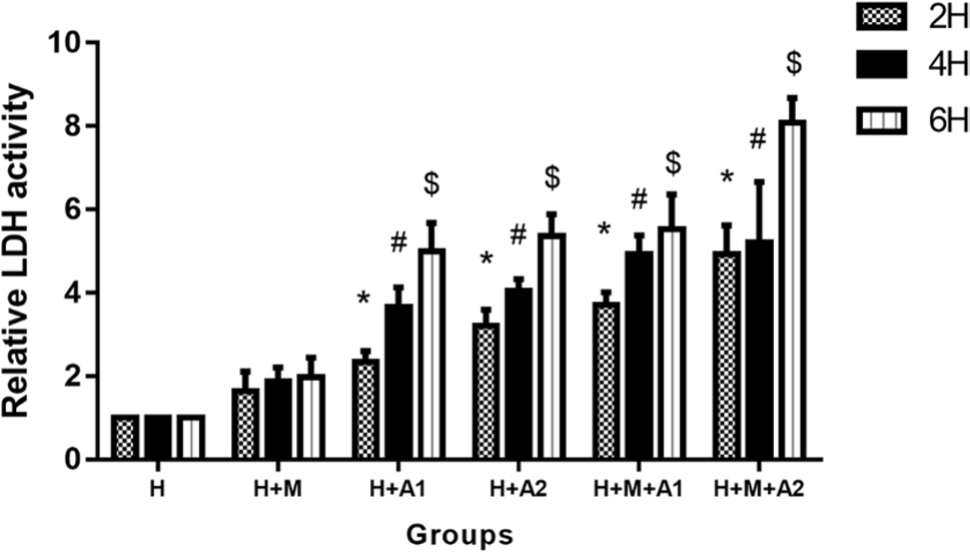
LDH level in the media of all groups for 2, 4, and 6 h. Each column represented the mean ± the standard error of the mean. *P < 0.05 vs. control at 2 h. ^#^ *P* < 0.05 vs. control at 4 h, ^$^ *P* < 0.05 vs. control at 6 h. The data were representative of at least three independent experiments

Figure 6 shows LDH release level in the media of all groups. There was a time- and concentration-dependent increase in LDH release by HK-2 cells. At 2 and 4 h incubation periods, LDH release significantly rose in all experimental groups except H + M group (*P* < 0.05). At 6 h incubation periods, LDH release increased more heavily than other time, and the difference between all experimental groups (expect H + M group) and the control were statistically significant (*P* < 0.05).

### BAX/BCL-2

**Fig. 7:**
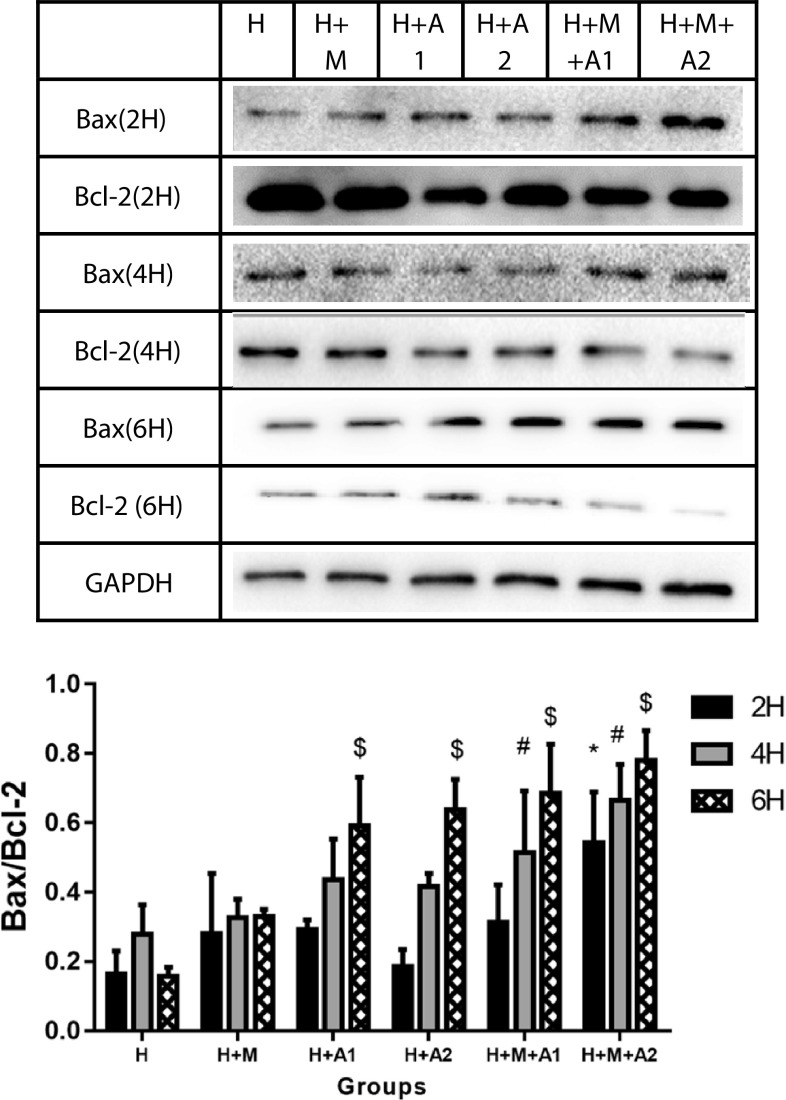
BAX/BCL-2 ratio in HK-2 cells of all groups at 2, 4, and 6 h, respectively. Each column represented the mean ± the standard error of the mean. ^#^ *P* < 0.05 vs. H group, 4 h. ^$^ *P* < 0.05 vs. H group, 6 h. The data were representative of at least three independent experiments

We assessed the apoptosis in HK-2 cells of all groups at 2, 4, and 6 h (Fig. 7). Hydroxyapatite (HAP) and macrophage cells increased BAX expression while inhibiting that of BCL-2 in a time-and concentration-dependent manner, which resulted in an increased BAX/BCL-2 ratio in the HK-2 cells. Only did the H + M + A2 group increase significantly compared with the control at 2 h incubation time (*P* < 0.05). At 4 h, there was significant difference in the H + M + A1 and H + M + A2 group compared with the control (*P* < 0.05). At 6 h, compared with the control, there was significantly increasing of BAX/BCL-2 ratio in all experimental groups except the H + M group (*P* < 0.05). As BAX is the promoting apoptosis protein, while BCL-2 is the suppressing apoptosis protein, the higher BAX/BCL-2 ratio means the more serious apoptosis. Our research results revealed that HAP could lead HK-2 cells to apoptosis in a time- and dose-independent manner; furthermore, the apoptosis can be aggravated by the M1 macrophage cells. In our observation window, the ratio of BAX/BCL-2 in H + M + A2 group at 6 h incubation time achieved the highest that meant the most serious apoptosis.

### Flow cytometry

**Fig. 8:**
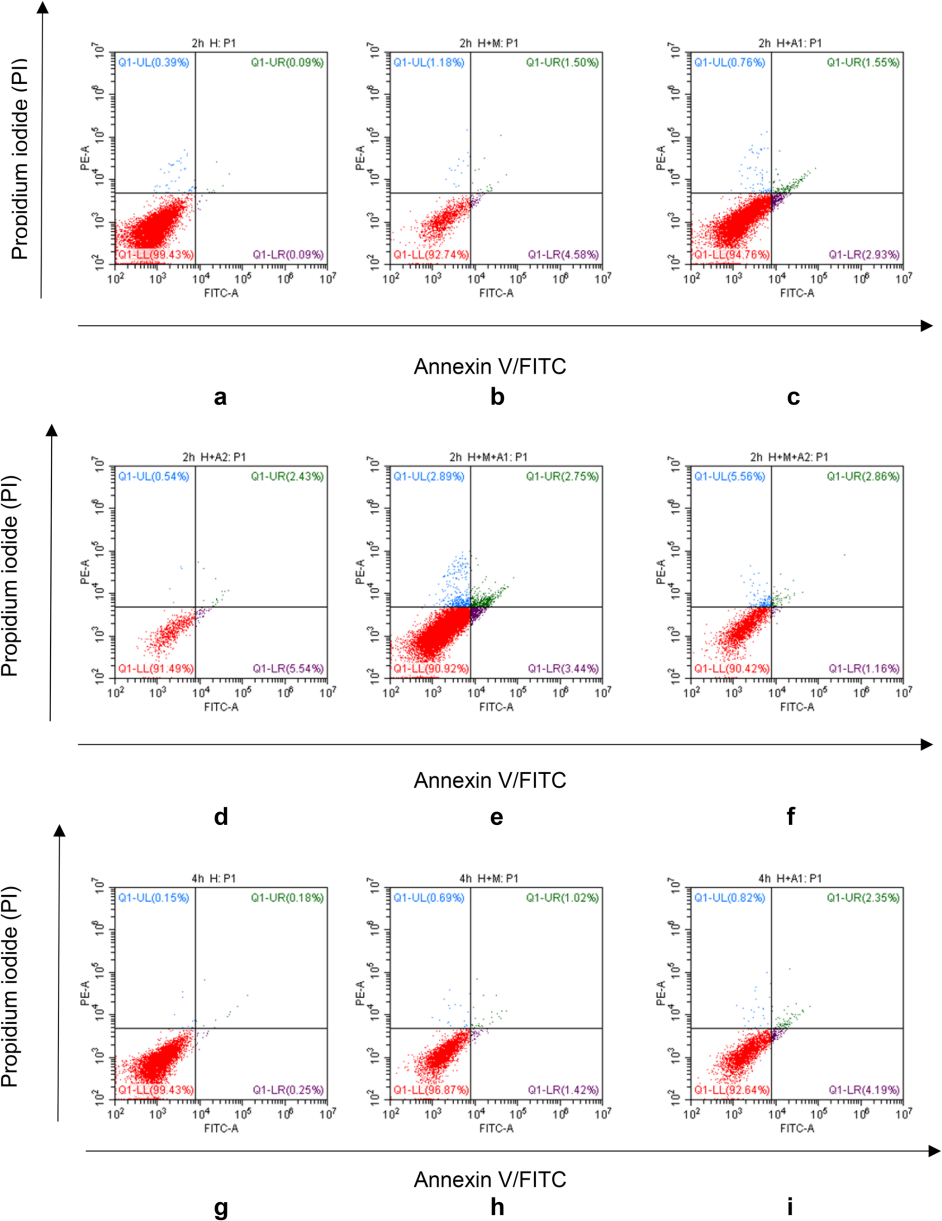

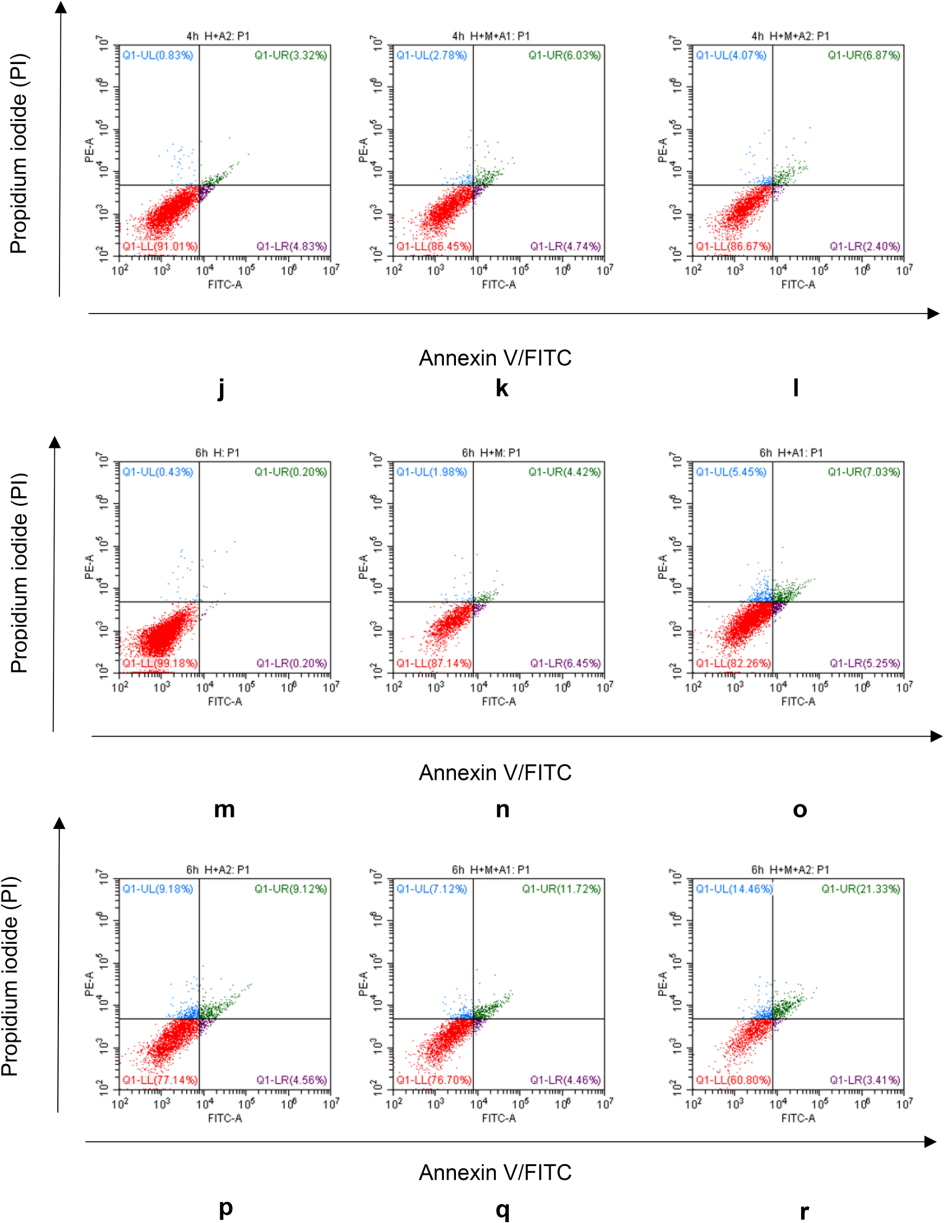
FITC-conjugated annexin V proteins and PI staining. After co-culturing with HAP and/or macrophage cells, HK-2 cells exhibited a significant progressive increase in annexin V^+^/PI^+^ staining

The apoptotic rate in our model increased in a dose- and time-dependent manner. From Fig. 8a–f, we find a small rise of apoptosis in our experimental groups at 2 h co-culture time. When came to the 4 h incubation time, apoptosis rate of HK-2 cells in experimental groups increased a little more clearly than 2 h, such as the apoptosis in H + M + A2 group had got 6.87%. When co-culturing for 6 h, the apoptosis of HK-2 cells became much more significantly than ever. Take Fig. 8m, n, p for example, HAP can cause apoptosis of HK-2 cells, and the macrophage cells can exacerbate the HAP-induced apoptosis of HK-2 cells. Moreover, the apoptosis rate of H + M + A2 group got the highest percentage of 21.33%, which provided further proof of our BAX/BCL-2 results in the western blotting analysis above.

### 4′-6-diamidino-2-phenylindole (DAPI) staining assay

**Fig. 9:**
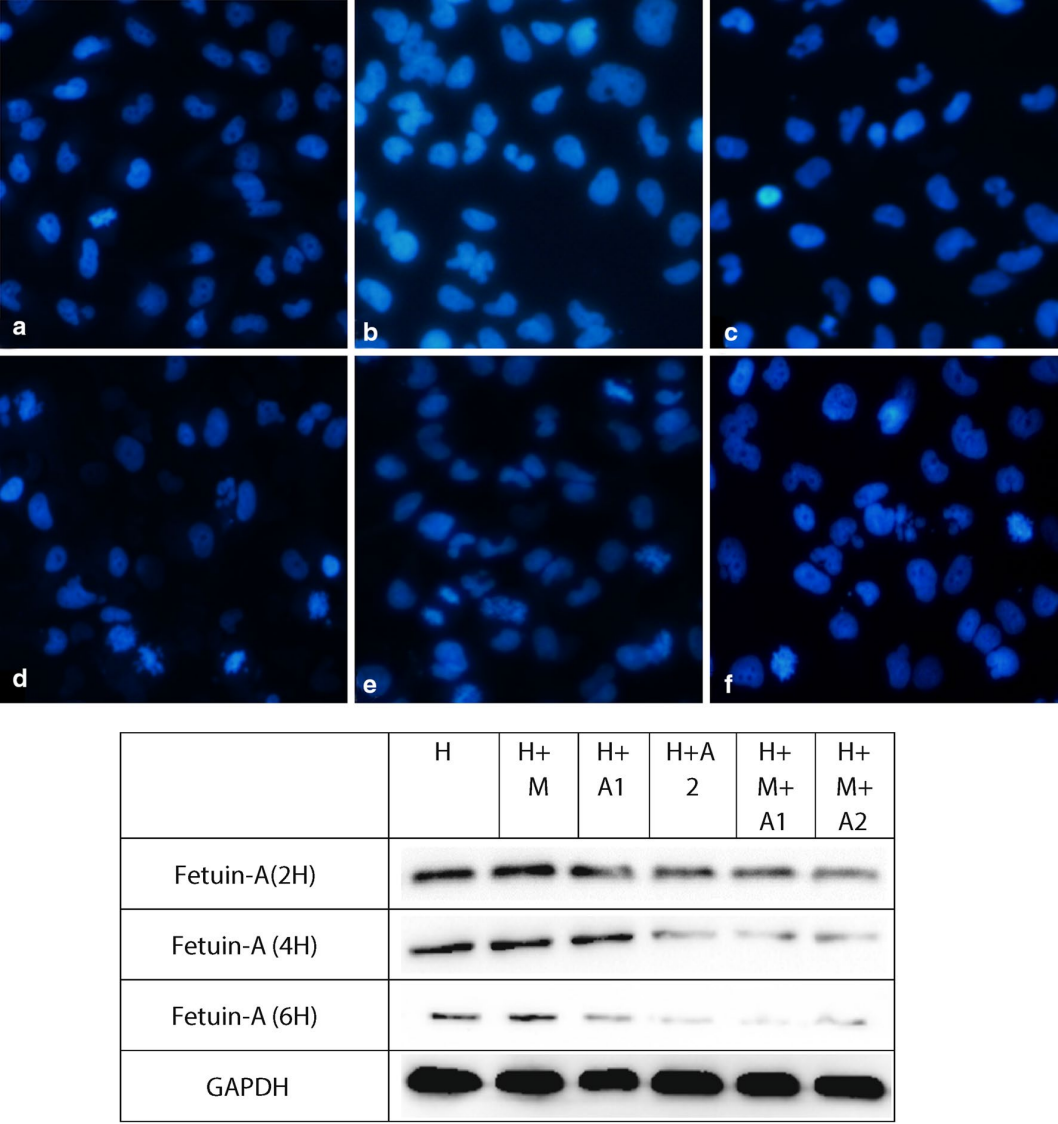
Original magnification, ×20. Scale bar 20 μm. Note: a, b, c, d, e, f represent H group, H + M group, H + A1 group, H + A2 group, H + M + A1 group, and H + M + A2 group

After co-culturing for 6 h, with the stimulation of macrophages and the increase of the concentration of HAP, apoptosis degree of each group aggravates gradually; moreover, the apoptosis degree of H + M + A2 group is most serious (Fig. 9).

### The expression of Fetuin-A

**Fig. 10:**
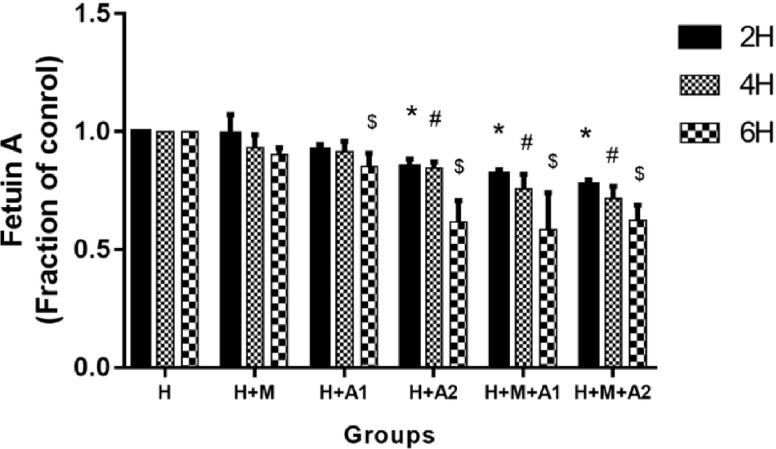
Expression of Fetuin-A in HK-2 cells of all groups at 2, 4, and 6 h, respectively. Each column represented the mean ± the standard error of the mean. *P < 0.05 vs. control at 2 h, ^#^ *P* < 0.05 vs. control at 4 h, ^$^ *P* < 0.05 vs. control at 6 h. The data were representative of at least three independent experiments

At 2 and 4 h incubation time, Fetuin-A decreased softly and a significant difference was observed at H + A2, H + M + A1, and H + M + A2 group (*P* < 0.05). When co-culturing for 6 h, more drastic decline of Fetuin-A in HK-2 cells had been found. Especially the expression of Fetuin-A in H + M + A2 group touched the bottom (*P* < 0.05) (Fig. 10). It declares that Fetuin-A in HK-2 cells decreases in a time- and HAP dose-dependent manner after HK-2 cells co-cultured with HAP, at the same time under the influence of macrophage cells, Fetuin-A in HK-2 cells drop even more.

## Discussion

Calcium oxalate crystals are a common component of urolithiasis; hydroxyapatite crystal is an important reason for the precipitation and condensation of calcium oxalate in the surface of renal papilla. As most of idiopathic calcium oxalate stone in the renal papilla adhesion area, Randall’s plaques (RP) located in the pulp loops descending thin section of the basement membrane. RP is thought to be nuclei for the formation of future stones, because RP is hypothesized to play a role in idiopathic calcium oxalate nephrolithiasis by eroding into the urinary space and acting as a scaffold for mineralization with calcium oxalate [7, 8, 10, 28]. RP’s main ingredient is apatite, which also includes calcium and magnesium phosphate [10]. Using high-resolution CT, Evan, A. P. and associates’ study [29] display that stones have grown over Randall’s plaque. What is more, Verrier, C, et al. recently reveal that RP contain various calcium and magnesium phosphates as well as apatite [10]. Therefore, we speculated that apatite has played a very important role in the development of urolithiasis.

Macrophage cells are the key cells that mediate innate and adaptive immunity. Macrophage cells can aggravate the inflammation, and the inflammation can exacerbate the formation of kidney stones [30]. Ruud de Water [11] investigates that macrophages and multinucleated giant cells are the major cells that encapsulate the interstitial crystals in both rat and humans. Eventually, M1 macrophage cells are thought to be pro-inflammatory [31]. Meanwhile, the phagocytosis of macrophages also cannot be ignored. In the animal models of calcium oxalate, Okada [14] found oxalic acid induced crystals formed in the tube cavity and then reached the interstitium, which disappeared in a few weeks. Ultrastructural analysis of kidneys shows that mononuclear macrophages are involved in the process. After calcium oxalate crystal-induced renal tubular epithelial cells to injury, the macrophages came to engulf and digest crystals with osteopontin, CD44, and fiber connection proteins mediating.

On one hand, macrophages may be transferred to the mesenchymal by swallowing CaO_*x*_ crystals [32] in the lumen to start the process of calcification of the renal nipple. During the inflammatory response of the crystalline macrophage, many inflammatory factors are secreted, causing chronic injury to the tissue and promoting the fibrosis of the kidney, and finally form the RP. On the other hand, after swallowing the CaO_*x*_ crystals, the macrophages induce the differentiation of epithelial cells into osteoblasts, which can provide apatite for the formation of RP [33]. The basic structure of Randall spot was formed by the deposition of renal papillary fibrosis and apatite. As a result, macrophages plays very an important role in regulation of RP-mediated renal calcium oxalate stone formation. It is not difficult to infer that there is an intertwined relationship between macrophage cells and urolithiasis.

Co-culture means two different kinds of cells culture together. At present, most co-cultured technique applied in bone cells and nerve cells. Cell co-culture system mainly through two ways: one is the direct co-culture system, namely two or more than two kinds of cells simultaneously or, respectively, inoculated in the same hole, different kinds of cells contact directly. The other one is indirectly co-culture system. The two or more than two kinds of cells were inoculated on different carrier, and then put the two carrier in the same culture. It can make different kinds of cells share the same culture system without direct contact. The advantage of cell co-culture techniques is that it can establish a co-culture system more like the body environment, making the internal environment consistent with external environment as far as possible, so that different kinds of cells are able to communicate and support with each other [12, 34–36]. Therefore, we co-culture HK-2 cells with HAP and/or macrophage cells; it can make up for the defects caused by study of usual single cells lines. Using this method of in vitro co-culture to explore the effects of HAP and macrophage cells to the expression of inflammatory factors and apoptosis in HK-2 cells of vitro co-cultured system, and how the macrophage cells regulate the influencing process.

As a negatively charged phosphorylated glycoprotein, OPN expressed in bone tissue, and expressed in the kidney, arterial vascular smooth muscle cell, urogenital tract, gallbladder, and other tissues. OPN showed a significant increase in renal expression levels in renal calculi rats [37], Langdon, A and Grohe, B investigated the interaction of OPN proteins and COM crystals by scanning electron microscopy and confocal microscopy, and found that the key control factor leading to the formation of stones was OPN [38]. By immunohistochemical method and transmission electron microscopy, Taguchi, K, and others found: OPN in RP expressed higher than the parts of renal tubular epithelial cells that do not contain RP [39]. These results revealed that OPN, as an important multi-functional protein, is associated so much with urolithiasis, the association is particularly prominent in OPN-mediated calcium oxalate crystals’ adhesion, deposition in the renal tubular epithelial cells.

However, it is controversial whether OPN promoted or inhibited the formation of stone. On one hand, Thurgood LA and other experiments in vitro found that OPN could inhibit the growth of COD in urine and adhere to renal epithelial cells, thereby inhibiting the formation of urolithiasis [40]. On the other hand, Hirose et al. found that in the early stages of stones when calcium oxalate crystals caused the damage of renal tubule epithelial cells, the adhesion of OPN could induce the formation of heterogeneous nucleation of stone crystals [41].

Based on the close correlation between OPN and urolithiasis, we also examined the expression of OPN in renal tubule epithelial cells at this study. In this study, we found that after the stimulation of HAP, the expression of OPN in HK-2 cells increased in a time- and dose-dependent manner, and macrophage cells can aggravate the increase of OPN in HK-2 cells. In our observation window, the H + M + A2 group show the highest OPN expression after 6 h co-culture. Our experimental results (shown in Fig. 4) are consistent with these findings [12, 41–45]. Therefore, we think that the increase of CCL-2 in culture medium and the up-regulation of OPN in renal tubular epithelial cells can cause a strong chemotactic effect on macrophages, aggravate inflammation [42–44], and exacerbate the process of crystal adhesion [12, 45]. In addition, the up-regulation of OPN also increases the heterogeneity of crystals and promotes the formation of stones, OPN combines with calcium phosphate to act as a matrix for stones, these matrix proteins also play a role in stopping to dissolve the calcium phosphate and inhibiting the accumulation and growth of crystals, and provide a form of aggregation and adhesion for free calcium ions in urine, therefore, oxalate or oxalate ions gradually formed nucleation on its surface and eventually formed calcium oxalate kidney stone [41].

As reported previously, renal epithelial cell injury and apoptosis facilitates crystal adhesion to cell surface, which serves as a key step in renal stone formation [17, 18]. Khan, S. R. reveals that renal epithelial damage may assist in the formation of Randall’s plaques [46]. Recently, Wang et al. find that COM crystals induced cytotoxicity and increased the lactate dehydrogenase (LDH) release in HK-2 cells [47]; Hu et al. also obtain similar results [48]. By the study of taurine interfered with calcium oxalate kidney stone model, some scholars find that the lower activity of mitochondrial antioxidant enzyme is related to the renal oxidative stress and the development of mitochondrial injury; meanwhile, the reason why taurine can protect the kidney may be related to the antioxidant effect of taurine itself [49]. When various causes lead to the activation of renal oxidative stress, it can lead to inflammation of renal tubular epithelial cells [50] and finally promote the accumulation of apatite deposits in the RP of renal tubular epithelial basement membrane. In addition, using the methods of optical microscope, transmission electron microscope, and Fourier transform infrared spectroscopy to analyze the biopsy specimens in patients with calculus, EVAN [51] discover a phenomenon that the epithelial cells at the attachment point of the stone were destroyed. The large accumulation of apatite in RP can change the physical and chemical environment of renal interstitium, calcify to RP, follow the flow of urine until to the kidney calices, and accumulate at the distal end small tube lumen. When the accumulation of HAP reaches a certain number, it can stimulate the hydrogenation of distal tubules, acidifying the urine, dissolving the calcium phosphate crystals at the end of the collection tube, releasing calcium and oxalate ions, forming calcium oxalate crystals, and finally cause the formation of calcium oxalate calculi.

At the same time, some scholars study the adhesion of the African green monkey renal epithelial cells with calcium oxalate and dihydrate calcium oxalate crystals before and after injury and the cellular response, they find that the adhesion and crystal concentration of calcium oxalate in the cells were positively correlated with the degree of cell damage. In the early stage, we also found significant calcium salt crystallization in renal tissue of renal calculi and showed obvious oxidative stress response due to cell injury [52], these indicate that oxidative stress and inflammatory injury are important reasons of the formation of urolithiasis.

Our current research is consistent with these conclusions. In our study, although macrophage cells cannot get through the membrane, they could interact with HK-2 cells by secreting inflammatory cytokines, such as C-C motif chemokine ligand 2 (CCL-2). From our ELISA results, we know that CCL-2 in the experimental groups increased in different degrees, HAP can stimulate the HK-2 cells to produce a certain amount of CCL-2, but the CCL-2 were mainly from the macrophages (Fig. 2). And by detecting the release of ROS, we find HAP can increase the fluorescence intensity of DCF in HK-2 cells, which up-regulate ROS in a time- and HAP concentration-dependent manner; macrophages can also exacerbate this enhancement effect. Therefore, we think that HAP can induce inflammation of HK-2 cells in a time- and HAP concentration-dependent manner, inflammatory factor released by macrophage cells can aggravate the inflammatory reaction through the microporous membrane, increase the expression of ROS, upgrade the oxidative stress reaction, and aggravate the damage of renal tubular epithelial cells. In addition, our LDH test results are further corroborating this phenomenon. LDH is an important enzyme in glycolysis, which exists in the cytoplasm of all tissues of the body and expresses higher in kidney. Cell damage may result in LDH release. And what we found was after co-culturing for 2, 4, and 6 h, the LDH activity increased in the experimental groups compared with the control group, besides the H + M group, the difference between the experimental group and the control group was statistically significant. It declares that after the stimulation of HAP, HK-2 cells damage broke, releasing LDH in a time- and HAP concentration-dependent manner, at the same time, macrophage cells can aggravate the increase of LDH. In our observation window, the relative expression of LDH was highest in H + M + A2 group after 6 h co-culture, indicating that the damage degree of HK-2 cells was also the most serious.

Our pre-study proved the cell apoptosis process can be activated after the renal tubular epithelial cells are damaged, this may increase the adhesion of crystal and the risk of stones formation [18].

From the western blotting analysis of BAX/BCL-2 (Fig. 7), we know the ratio of BAX/BCL-2 in the H + M + A1 group was higher than H + A1 group after 6 h co-culture; therefore, we believe that macrophages can aggravate the apoptosis of renal tubular epithelial cells. More importantly, the results of our Flow Cytometry and DAPI staining also confirm that HAP and macrophages can induce and aggravate the damage of HK-2 cells. Using Flow Cytometry to detect the apoptosis rate of HK-2 cells, we find in the third quadrant, the positive rate of FITC and PI increases in different degrees compared with the control group (Fig. 8). This shows that in our in vitro co-culture model, HAP can increase the apoptosis of HK-2 cells in a time- and HAP concentration-dependent manner, and macrophages can increase the apoptosis of HK-2 cells induced by HAP. In addition, our DAPI staining results (Fig. 9) also show that the apoptosis rate of HK-2 cells is gradually increasing, which also confirms that macrophages have a certain regulatory effect on the damage of HK-2 cells induced by HAP. Therefore, it is not difficult to speculate that after HAP stimulates the renal tubular epithelial cells, the HK-2 cells are damaged and apoptotic, which is not only possible to expose phosphatidyl-serine (PS) to the cell surface [53, 54], and may also express kinds of crystal adhesion molecules such as HA, OPN, CD44, membrane protein II, glycoproteins containing sialic acids, and nucleolin-related proteins (NRP), these molecules have negative charge, which either can affect the oxalic acid root (O_*x*_^2−^) role of calcium oxalate by hydrogen, or bond with calcium oxalate crystals by electrostatic interactions, thus greatly enhance the ability of the cells to bond to the crystal after cell damage. The damage of HAP to the renal tubular epithelial cells was further aggravated by the inflammatory factors secreted by macrophages, resulting in more calcium oxalate crystals attached to the renal tubular epithelial cells.

Fetuin-A, which is in sites of vascular calcification, has long been considered a powerful inhibitor of ectopic calcification and inflammation [55–60]. HAP acts as the plaque’s main component in patients with kidney stones, and Fetuin-A also conducts to obtain a high affinity for HAP [10]. Marked extra-bone calcification has been remarkably reported in Fetuin-A knockout mice [20]. Normally, it seems that the kidney should have increased the expression of Fetuin-A to protect from the progress of inflammation induced by oxidative stress and overproduction of renal calcium. Nevertheless, interestingly, the previous studies [21, 52] imply that lower Fetuin-A protein level in urine and renal tissue may be one of the hazards for urolithiasis. Daveau, M regards Fetuin-A as a negative acute-phase protein which can be down-regulated by cytokines [61]. Li et al. [62] also found that the early inflammatory cytokines could inhibit the expression of Fetuin-A. Our results were similar with these studies, but not with Aksoy [63]. As shown in Fig. 10, we detected the different degree of declining expression of Fetuin-A at different incubation period compared with the control, following with the up-regulation of inflammatory cytokines. Especially, the expression of Fetuin-A in H + M + A2 group touched the bottom at 6 h. These findings tell us after being stimulated by HAP, the HK-2 cells can down-regulate to express Fetuin-A in a time- and HAP concentration-dependent manner. At the same time, macrophages secrete a large number of CCL-2 which pass through the microporous membrane and aggravate the inflammatory response; this may be associated with the further decline of Fetuin-A in HK-2 cells. Some scholars find Fetuin-A has inhibitory effect on ectopic calcification and the deposition of calcium salt caused by apoptosis and inflammation induced by oxidative stress (OS) [55–57]. Consequently, we confirm that Fetuin-A may possibly inhibit OS. On the contrary, excessive OS induced by the increment of apoptosis, the up-regulation of OPN in HK-2 cells, the increased CCL-2 in medium mainly secreted by macrophage cells may possibly consume much of Fetuin-A, but the ability of the cell to secrete Fetuin-A is limited. When it exceeds the cell’s secretion capacity, the expression of Fetuin-A shows a downward trend. Besides, apoptosis can also result in declining the ability of the HK-2 cells secreting Fetuin-A. However, this is just our supposition, further studies are needed to clarify the reasons of the decline of Fetuin-A.

In conclusion, in our in vitro co-culture model study, the stimulus of HAP can set off an inflammatory response in HK-2 cells, lead to apoptosis, increase the expression of OPN in HK-2 cells, and decline the expression of Fetuin-A. Especially, in the macrophage-renal tubular epithelial cell-HAP co-culture system, oxidative stress, the increase of inflammatory factor CCL-2 in renal tubular epithelial cells and cell apoptosis were more obvious, OPN went up more and Fetuin-A declined more dramatically. This shows that macrophages have a certain regulatory effect on the expression of HAP-induced inflammatory correlation factors in HK-2 cells. Therefore, we think that, on one hand, once the renal tubular epithelial cells occur inflammatory damage or trigger apoptosis process, not only does the release of reactive oxygen species increase, inflammatory factors and OPN protein secretion increase, intensifying the inflammation, causing the crystal adhesion, deposition, and heterogeneous nucleation, but also probably consume large amounts of Fetuin-A through oxidative stress and inflammatory response, inhibit the ability of HK-2 cells to secrete Fetuin-A, and decline the expression of Fetuin-A; however, the drop of Fetuin-A will decrease the body’s ability of inhibiting the ectopic calcification, eventually promote the formation of stones. On the other hand, CCL-2 and OPN can also have a chemotactic effect on macrophage cells, which enhance the migration ability and phagocytic ability of macrophage cells. After swallowing the CaO_*x*_ crystals, macrophage cells can increase the expression of NAPDH oxidase in HK-2 cells, aggravate the inflammation, and induce the necrosis and denaturation of renal tubular epithelial cells; the basement membrane of renal tubular epithelial cells was exposed, thus promoting the formation of Randall plaques and even urolithiasis.

In consequence, if we can stop the release of the CCL-2, OPN, and ROS, inhibit the oxidative stress and inflammatory injury and apoptosis of renal tubular epithelial cells caused by crystal reaction, and learn more about the regulation mechanism of Fetuin-A, it will help us to understand more about the formation mechanism of Randall’s plaques, and may provide new ideas for further elaboration of the pathogenesis of urolithiasis.

However, given that this was only an in vitro study and we did not confirm our idea from animal models, it is difficult to determine whether our findings are the most representative of explanation for renal stone formation in human beings. In addition, we should not have been simply excluded the other kinds of crystals in renal interstitium from our study, like calcium oxalate. Moreover, we did not investigate the effect of inhibiting ROS generation on the expression of Fetuin-A. Furthermore, it was absent of direct evidences whether the cell damage resulted from the HAP and macrophage cell-induced inflammation can promote crystal adherence and deposition in HK-2 cells. Eventually, further studies are also needed to determine whether there is some kind of mechanism that can regulate the expression of Fetuin-A in kidney and can protect renal tubular epithelial cell against apoptosis. We need further study to compensate for our weaknesses.

## Electronic supplementary material

Below is the link to the electronic supplementary material.

Supplementary material 1 (XLSX 17 KB)
